# Spatializing Air Conditioner and Evaporative Cooler Ownership, Use, and Inequality across 400+ Districts Pan-India: Unpacking Social, Environmental, and Urbanization Correlates

**DOI:** 10.1021/acs.est.6c05101

**Published:** 2026-10-07

**Authors:** Neha Agarwal, Anu Ramaswami

**Affiliations:** Department of Civil and Environmental Engineering and M. S. Chadha Center for Global India, 6740 Princeton University, Princeton, New Jersey 08540, United States

**Keywords:** urban cooling futures, electricity use, inequality, social-environmental-urbanization modeling (SEU-modeling)

## Abstract

Cities/districts in rapidly urbanizing, developing nations, such as India, are grappling with extreme heat and a potential tripling of electricity use for cooling by 2050, estimated by global models. Yet, little is known about the adoption of air conditioners (ACs) versus evaporative coolers (ECs), nor inequality in their use across income groups and climate regions, at the district-level pan-India with diverse social, environmental, and urbanization (SEU) features. This paper contributes a new systems-level method to spatialize district-level household-average AC and EC ownership, and inequality in their electricity usage, across 400+ districts pan-India, combining SEU-based analyses of large-scale household surveys with Random Forest Modeling and Propensity Score Matching. Key findings are (1) National household-average ownership in 2022 of EC-only, AC-plus-EC, and AC-only is 18%, 3%, and 4% respectively, with high-income, high-humidity, urban districts showing 10× AC ownership, e.g., 32% in Chennai, reflecting urban futures; (2) Our AC-versus-EC model, for the first time, demonstrates dependence on both local humidity and air temperature in addition to income and other social variables with high predictive accuracy (kappa = 0.76); (3) Differential electricity use for cooling varied by income with highest-income homes consuming Δ = 178 kWh/capita/year for ACs and 56 kWh/capita/year for ECs compared to homes without cooling appliances nationally, while mid-income homes consumed 56% and 64% less for ACs and ECs, respectively; these patterns varied across hot-humid, hot-dry, and composite-climate regions across India. These results highlight the low penetration of ACs and their highly frugal, unequal use in India. Together, they lay the foundation for urban futures modeling at local scales pan-India, incorporating highly heterogeneous and massive anticipated changes in urbanization, income, and climate, with methods translatable globally.

## Graphical abstract

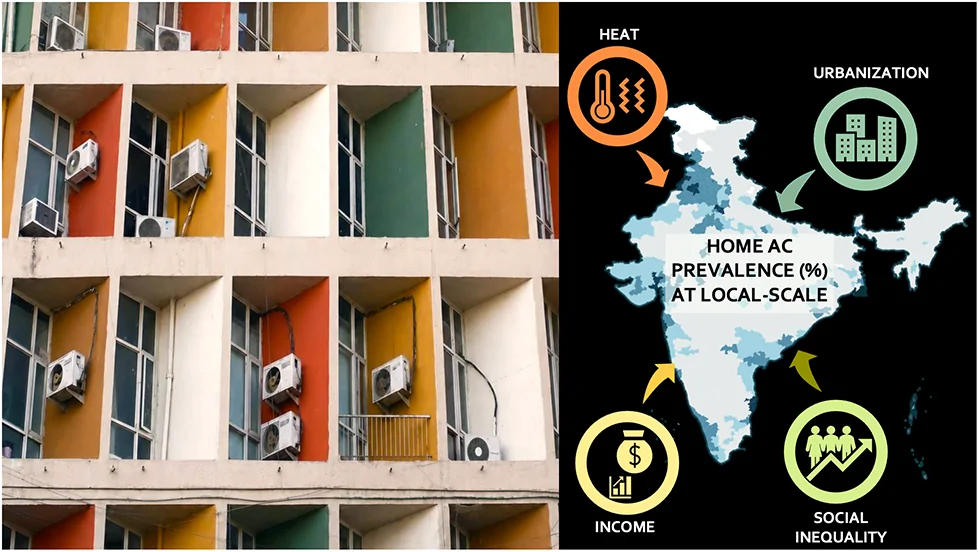

## 1 Introduction

Half the world’s population is expected to face extreme heat by 2100.^1^ The Urban Heat Island Effect (UHIE) further exacerbates extreme heat in urban areas, as greater concretization raises air temperatures relative to rural areas. Beyond air temperature, factors such as relative humidity and solar radiation also affect heat stress.^2^ Indeed, by 2100, >3 billion people are expected to live in urban areas with high humid heat stress, with >1 billion in South Asia alone.^3^ Consequently, cities globally, including those in India (e.g., Delhi, Chennai), are undertaking heat resilience planning,^4,5^ while ∼1000 cities worldwide are engaged in decarbonization planning^6^ that requires local electricity use data. Global models estimate a tripling of electricity demand for cooling by 2050 globally, with India one of the top three contributors to this growth.^7^ However, although urban areas have now become a key arena for climate action^8,9^ and the district level holds high administrative significance in India,^10^ there are foundational data gaps at the local level for India’s 1.4 billion people living in 600+ diverse districts. Specifically, data on cooling appliance ownership, use, and inequality at the district level are poorly quantified pan-India. Currently, this data is largely available only at the national level, particularly in developing countries^11–13^ or in case studies of a few districts.^14,15^ Our paper addresses this gap by developing a first Social-Environment-and-Urbanization (SEU)-based^16^ model to estimate cooling appliance ownership, use, and inequality at the district scale across India.

Air conditioners (ACs) and evaporative coolers (ECs, hereafter) are the prevalent cooling technologies in India.^14^ Homes may prefer ECs to ACs due to their lower capital and operating costs. The effectiveness of ECs is also expected to vary across India’s diverse climates, with higher effectiveness in the drier North and lower in the humid coastal South. However, even in the North, effectiveness can decline as regional humidity increases.^17^ Despite their current prevalence and potential future ineffectiveness, particularly affecting low-income households, ECs are absent from most studies of cooling transitions in India. Therefore, investigating AC-versus-EC ownership as a function of local SEU attributes is important. Unlike choices for mobility, i.e., cars-versus-motorcycles,^18^ no prior work has investigated AC-versus-EC ownership for India. Ahn & Uejio (2025) have recently developed a Random Forest model for predicting types of cooling appliances at local scales using a private high-resolution real estate data set for the US.^19^ The method has promise; however, such data are lacking in many developing and rapidly urbanizing economies, where household surveys must be analyzed to glean similar information. India’s landmark household survey on consumption expenditures systematically surveyed more than 260,000 homes across India in 2022–23, but reported on combined ‘AC or Cooler’ ownership, resulting in a large data gap on who owns AC versus EC among India’s 1.4 billion people living in 600+ districts.

Beyond ownership, investigating the associated electricity consumption of ACs and ECs across income groups and regions is also important for two reasons. First, to assess whether cooling appliances translate into sustained use, which ultimately determines vulnerability to extreme heat, recognizing that low-income homes often use less electricity than high-income homes.^20^ Second, to develop context-specific estimates of the additional electricity demand for cooling that regional power utilities must plan for. Prior national studies estimate that AC-ownership increases households’ electricity consumption by 36% on average across countries,^13^ but contextual pan-India estimates of this differential effect, with an explicit focus on income inequality, are lacking.

To address these foundational knowledge gaps at the local level, the overall goal of this paper is to advance data and methods on cooling ownership, use, and inequalities at policy-relevant local scales. The specific objectives of the paper are

1. Develop a social-environment-and-urbanization (SEU)-dependent model for AC-versus-EC ownership at the household-level for India.
2. Spatialize the household-level model across >600+ districts of India, consolidated into 421 district-equivalents based on data availability.
3. Quantify inequality in differential electricity consumption associated with AC- or EC- ownership by income groups, pan-India, and in major climate regions.

Our methodology is relevant not only to India but also to other developing nations in Africa and South/Southeast Asia that are experiencing simultaneous urbanization, economic growth, and extreme heat. Our methodological innovation lies at the systems-level, wherein we demonstrate how disparate SEU data can be brought together to inform AC-ownership and use at a granular scale. Such integrated SEU analyses are at the frontier of sustainable urban system science.^16,21^ Such system models represent a new paradigm in environmental modeling, where integration of environment (climate), infrastructure conditions, urbanization, in addition to socioeconomic status provides vastly different results (compared to prior income-only models) and captures granularity important for urban-scale energy demand futures modeling. Such improved estimates of current and future fine-scale AC-ownership and electricity demand projections will be essential for cost-effective electric utility and energy system planning in developing nations. Such data are unavailable not only in the developing world but also in the EU and other developed countries with sparse AC-ownership.^19^ Thus, our overall SEU methodology is original, relevant, scalable, and essential for any fine-scaled attempt at modeling energy demand in a warming world.

## 2 Methods

Our methodological framework (see Figure 1) involves five steps:

**Figure 1:**
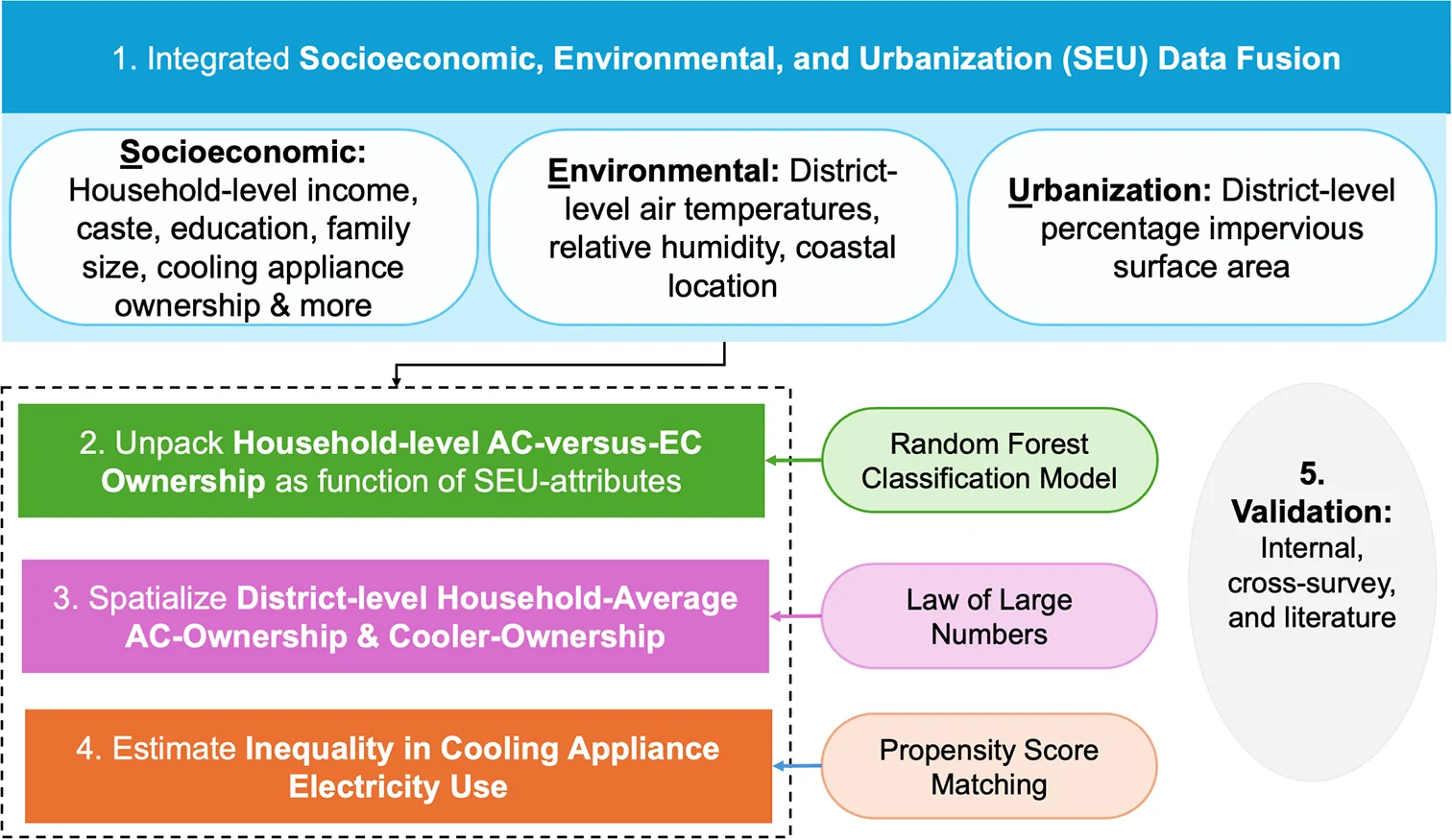
Five-step methodological framework developed in this study. AC: air conditioner, EC: evaporative cooler.

First, create an integrated SEU data set that includes household-level cooling appliance ownership, electricity consumption in physical units (kWh), and household- and district-level SEU attributes, by combining multiple data sets, anchored upon India’s landmark household consumption expenditure survey.

Second, unpack AC-versus-EC ownership, which is reported together as ‘AC or Cooler’ ownership, i.e., cooling appliance ownership, by developing a Machine Learning-based classification model to predict household-level AC-versus-EC ownership. The classification model provided insights into which household- and district-level SEU attributes inform AC-versus-EC ownership.

Third, spatially characterize district-level household-average AC-ownership and EC-ownership (% households owning ACs or ECs) and use (kWh/capita) across districts in India, incorporating the dependence of these variables on SEU attributes, such as local household income and asset ownership, caste, urbanization, relative humidity, and air temperature, derived from the previous step.

Fourth, estimate inequality in cooling appliance use using propensity score matching.

Lastly, validate model results by comparing them with other surveys in India and by contextualizing them with existing literature. Each of these steps is described in the following section.

### 2.1 SEU (Social-Environmental-Urbanization) Data Integration

Our paper integrates **S** ocial (household-level data on income and asset ownership, caste, gender of household head, education, employment, household size), **E** nvironmental (district-level data on air temperature and relative humidity), and **U** rbanization (district-level data on percent impervious surface area) across 600+ districts of India. Two household survey data sets have been utilized in this paper: the Government of India’s National Sample Survey on Household Consumption Expenditures (HCES),^22^ which surveyed >260,000 homes in 2022, and the privately run Consumer Pyramids Household Survey 2022–2023 (CPS), which surveyed >120,000 households.^23^ The Global Human Settlements Layer data set on Built-up Surfaces^24^ was used to obtain district-level urbanization estimates, and ERA5 data sets^25^ were used for air temperature and relative humidity.

### 2.2 Household-Level AC-versus-EC Ownership

India’s HCES provides data on household electricity consumption in physical units, critical to our analysis, but does not report ownership of ACs and ECs separately. The private CPS provides data on household ownership of ACs and ECs separately but lacks data on electricity consumption in physical units. Therefore, the two data sets were bridged using a Machine Learning-based classification model trained on the CPS data to predict AC-versus-EC ownership. The model was applied to the HCES data set to obtain decoupled AC-versus-EC ownership, in tandem with each household’s electricity consumption in physical units.

Three Machine Learning approaches, i.e., LightGBM, Random Forest, XGBoost, and traditional Multinomial Logistic Regression, were explored in this study to decouple AC-versus-EC ownership. The classification model predicts three classes of ownership among homes reported to own any type of cooling: ‘EC-only’, ‘AC-plus-EC, and ‘AC-only’. Household-level and district-level SEU attributes were used to inform the classification model’s predictions.

Detailed SEU attributes included were: SOCIAL: (1) household-level income represented by its proxy MPCE and assets (ownership of car, motorcycle, computer), (2) household-level ownership of electricity appliances: ownership of refrigerators, washing machines, (3) household-level building characteristics: building materials for the house roof and wall, access to a private toilet, and (4) household-level sociodemographic characteristics: gender of household, household members >60 years of age, married couples, college-educated members, religion, caste, household size; whether the household resides in an urban or rural area. ENVIRONMENTAL district-level features included (1) cooling degree days (estimated from district average air temperatures at a threshold of 22°C), (2) mean relative humidity, (3) number of days with high heat index (based on the classification from the US Occupational Safety and Health Administration), and (4) its coastal or interior location. URBANIZATION features included district-level urbanization quantified as the percentage of impervious surface area.

The Social (S) features were selected based on insights from prior studies in India and other developing nations on the importance of income, assets, as well as female-headed households and caste-based inequality in impacting electricity use.^20^ The relevant Environmental (E) features were selected based on prior global literature that identified air temperature (represented as cooling degree days),^13^ as well as the authors’ own hypothesis that in developing nations, relative humidity will also play a role, given the rise of humid heat in Asia and Africa^3,26^ alongside urbanization and the Urban Heat Island Effect.^27^ The overarching hypothesis in this paper is that all three types of SEU attributes will together shape the evolution of cooling appliance ownership and its usage. Data sources for the SEU attributes, along with the scales of data granularity, are summarized in Table SI-1.

#### 2.2.1 Analytical Approach

The subset of households with any type of cooling served as the basis for the model development. This subset was partitioned into training (80%), calibration (10%), and test (10%) sets using stratified sampling to preserve the class distribution across all splits, with the calibration set held out exclusively for post hoc probability calibration and the test set reserved for final model evaluation. Model performance was assessed using standard class-wise and overall metrics, including precision, recall, accuracy, area under the curve (AUC) score, and F1 score.

The uncalibrated Random Forest Model was selected as the final model because it achieved a good balance between identifying households in the two minority cooling classes, ‘AC-only’ and ‘AC-plus-EC’, and overall model performance, as discussed in Section 3.1. This SEU-dependent Random Forest classification model was applied to the HCES data set, yielding decoupled AC-ownership and EC-ownership at the household level.

### 2.3 Pan-India District-Level Household-Average AC-Ownership and EC-Ownership (% Households)

Our method leverages the Law of Large Numbers to spatialize averages that can be computed from household survey data, e.g., district-level household-average AC-ownership and EC-ownership, or other central tendencies such as district-level household-average electricity use, as has been demonstrated effectively previously for Indian districts (*n* = 640) in 2011.^28,29^ For example, for continuous variables, such as electricity use per home, the error in the district-level household-average electricity consumption (with *n* ≥ 60 surveys per district) is expected to be ∼4% at 95% Confidence Interval.^30^ Further, the current 2022 electricity use, computed bottom-up in this paper from the survey data (based on district-level averages with *n* ≥ 60 surveys in each district), compares well with utility data^31^ (see Figure SI-6). This shows that the bottom-up survey-derived electricity use data are physically meaningful and align well with utility data.

For categorical variables as well, such as ownership of ACs and ECs, *n* ≥ 300 household survey samples were selected as a minimum threshold based on principles of survey statistics and analysis. This yields a maximum theoretical Margin of Error of 5.7 percentage points at 95% Confidence, at a probability of ownership (of AC or EC) of *p* = 0.5. Since not all districts had 300 or more samples, adjacent districts with insufficient samples were clustered to obtain districts (or district-equivalents) with more than 300 samples. Clustering was implemented only for spatially contiguous districts with less than 300 household survey samples using a graph-based algorithm in Python, which was then manually inspected in QGIS and readjusted to ensure the minimum possible clustering while ensuring the minimum threshold of 300 household survey samples was met across all district-equivalents. Overall, 144 new district-equivalents were created, with each district-equivalent combining two to six districts with *n* < 300 household survey samples, contributing to a total of 421 effective districts (i.e., original districts plus the constructed district-equivalents) across India. Socioeconomic, climate, and urbanization (SEU) features of the original district were compared with the combined district-equivalent. The average difference across districts was observed to be 12% for income (with 85 of 354 districts exceeding 15% difference); 21% on average for cooling degree days (with 85 of 354 districts exceeding 15% difference); and 0.4 percentage points for urbanization (with only 1 of 354 districts exceeding 15 percentage points). Thus, overall, the vast majority (75%) of districts that were merged to form a district-equivalent had similar SEU attributes.

Within each original district or merged district-equivalent, the household survey sampling (*n* ≥ 300) is assumed to be representative of that unit, given India’s HCES sampling methodology that aims for representativeness through a multistage stratified sampling strategy that allocates samples from the state-level down to each state’s urban/rural populations and further to each subsettlement sampling unit with population-proportional allocation maintained at each stage.^22^ Such representativeness was further affirmed by examining whether the histogram of the Monthly Per Capita Expenditures (MPCE) of the surveyed homes followed a near-log-normal distribution, which has been observed by economists, based on empirical data from several developing nations.^32,33^ Illustrative examples in Figure SI-6 show that the distribution of MPCE of surveyed homes in the 421 district-equivalents (all with *n* ≥ 300 household surveys) followed a near-log-normal distribution, affirming representative sampling.

### 2.4 Inequality in AC and EC Electricity Use by Income and Climate

Our study uses HCES household survey data because it reports households’ electricity use in physical units (kWh/month), already validated with utility data^31^ (see Figure SI-7). These data were evaluated using propensity score matching (PSM) to quantify the inequality in electricity consumption associated with cooling appliance ownership across income groups, pan-India and within India’s three climate regions (hot-humid, hot-dry, composite-climate) based on the National Building Code (NBC) 2016 classification.^34^ The corresponding hours of cooling, or AC or EC use, were inferred from this differential electricity consumption.

In the PSM analysis, household ownership of a given cooling appliance (AC or EC) was considered as the treatment group and nonownership as the control. Treatment and control groups were carefully selected to ensure that they were similar in all key SEU features, except for the ownership of the cooling appliance itself. Thus, for example, among the highest-income homes, similarly high-income homes were compared, with similar asset ownership, education levels, etc., except that the treatment group owned ACs, while the control group did not. The PSM analysis was conducted nationwide (pooling all survey data) and within India’s major climate regions to obtain large sample sizes for the treatment and control groups. Such an analysis was difficult to conduct at the district level pan-India, due to a small set of robust treatment and control matches owing to smaller sample sizes within each income group.

To create treatment and control groups, a logistic propensity score model was fitted on observed household covariates, including income (MPCE, car, and motorized two-wheeler ownership as proxies), electricity appliance ownership (washing machine and refrigerator), household size, caste, urban or rural status, and district-level cooling degree days; differences in the state and survey panel were also controlled for. Each treated household was matched with replacement to its nearest control neighbor within a caliper of 0.2 standard deviations of the logit propensity score, restricted to the common support region. The different electricity consumption associated with cooling was computed as the mean difference in per-capita residential electricity consumption between matched treated and control homes. 95% confidence intervals were estimated using bootstrapping (*n* = 1000), as PSM without bootstrapping provides only a point estimate.

The balance between the treatment (AC-ownership and EC-ownership) and control groups (no cooling appliances) was assessed using standardized mean differences (SMD).^35^ Results are reported only for estimates with at least 30 matched treated samples, significant effects, and maximum |SMD| of <0.4 across all covariates. Overall, good balance was achieved between treatment and control groups, with actual |SMD| < 0.1 across most covariates. In all specific analyses reported, across income quartiles nationally and across India’s three climate regions, the matched sample sizes exceeded *n* = 78 and yielded statistically significant comparisons (*p* < 0.05).

Further, Rosenbaum bounds sensitivity analysis^36^ was conducted to evaluate the robustness of results to unobserved confounders. Sensitivity is assessed with the tipping-point odds ratio (Γ), which represents how strong an unobserved confounder’s effect would need to be for the results to lose statistical significance at *p* < 0.05; Γ ≥ 1.1 represents analysis is robust to unobserved confounding variables.^37^ The Rosenbaum bounds sensitivity analysis was conducted on without-replacement PSM matches and yielded Γ = 1.28–1.96 for PSM analyses of AC electricity use across region- and quartile-specific analyses, indicating the robustness of the analysis to unobserved confounding variables. PSM analyses of EC electricity use were also robust (Γ = 1.10 to 1.67), with the only exception being Q1 of the composite-climate region with a Γ = 1.06.

### 2.5 Validation of Household-Level AC-versus-EC Model: Within-Model Validation

Our Random Forest classifier performed very well in internal model validation (kappa = 0.76), showing the best performance among all four classifiers evaluated on the held-out test set (see Table SI-2). It also achieved the best balance between identification of households in the two minority cooling classes, AC-only and AC-plus-EC, and overall model performance, with recalls of 0.87 and 0.81, respectively.

### 2.6 Validation with Independent Survey Data

Overall, the model predicted national-average ownership of ‘EC-only’, ‘AC-plus-EC’, and ‘AC-only’ at 17.6%, 3.2%, and 3.6%, respectively. The absolute Standard Error (SE) in district-level household-average AC-ownership and EC-ownership (%) was <3 percentage points and depended on the number of household survey samples within a district. For example, one of the highest SE in AC-ownership was 1.8 percentage points in a district with AC-Ownership of 12% and *n* = 324, and one of the highest SE in EC-ownership was 2.9 percentage points in a district with EC-ownership at 46% and *n* = 302. SEs were much lower in all other districts due to larger sample sizes. The district-level household-average AC-ownership and EC-ownership percentages aligned well with a separate survey, MICS 2019, with a different sampling strategy and conducted in a different year, with an *R* ^2^ of 0.86 for AC-ownership showing broad agreement (see Figure SI-9).

### 2.7 Validation of Inequality in Cooling Appliance Electricity Use

Robustness of the national-level PSM analysis was further evaluated by comparing electricity consumption between the hot season and the rest of the year among homes with ACs and ECs, yielding similar results (see Table SI-3). Further, these estimates were compared with estimates in the literature and showed reasonable agreement (see Table SI-4).

These validations and uncertainty analyses showed that the model results are robust.

## 3 Results

Our results are in three sections. Section 3.1 presents the results of our SEU-dependent household-level AC-versus-EC classification model, highlighting which SEU attributes influenced preference for AC-versus-EC. Section 3.2 presents district-level household-average AC-ownership and EC-ownership (%) across 421 districts pan-India with varying incomes, urbanization, and climates. The model’s strength in representing local SEU attributes is demonstrated by contrasting AC-ownership profiles as a function of income across six diverse districts that represent urban/rural, high/low income, and climate effects. Section 3.3 addresses inequality estimates of household electricity consumption associated with ownership of cooling appliances across income groups, nationally and in warm-humid regions. The differential electricity consumption for cooling is quantified in terms of a cooling appliance’s hours of operation.

### 3.1 Household-Level AC-versus-EC Ownership

Household income (or monthly per capita expenditure, MPCE, as its proxy) shows a strong positive association with preferential AC-ownership relative to EC-ownership, as expected for a rapidly urbanizing developing economy. Our model is the first to demonstrate the modulating impact of multiple SEU attributes other than household income, such as household caste, level of household energy services (i.e., ownership of other electrical appliances, particularly washing machines), and local relative humidity, *as well as* air temperatures, on AC-versus-EC ownership (Figure 2). Prior studies have evaluated only a subset of these SEU attributes and only in the context of AC-ownership; e.g., see the Introduction section; among which only one prior study has revealed relative humidity correlations with AC-ownership in addition to cooling degree days. This study finds an association of both relative humidity and air temperature alongside multiple socioeconomic factors on cooling appliance ownership, unpacking AC-versus-EC ownership. The SEU-informed AC-versus-EC ownership model shows superior performance (kappa = 0.76) compared to an income-only model (kappa-0.3). Figure 2 shows that as household incomes increase, the preference for ECs declines in favor of ACs, indicating that ECs are an inferior asset relative to ACs.

**Figure 2:**
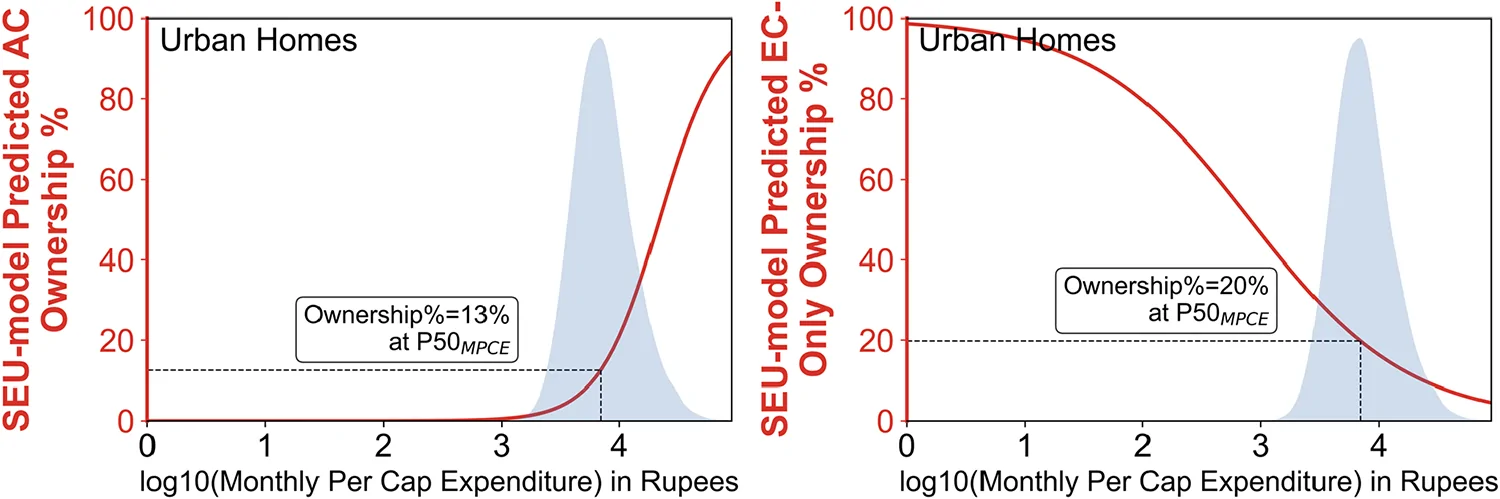
S-shaped probability curves based on the social-environmental-urbanization (SEU) model for (a) AC and (b) EC-only ownership for urban homes. MPCE represents monthly per capita expenditure in rupees. The distribution of MPCE is shown in gray. Predicted ownership of AC and EC-only among urban homes at median urban MPCE is shown in text boxes.

The role of household- and district-level SEU attributes in influencing the preference for AC-versus-EC is demonstrated with Shapley Additive Explanations (SHAP) plots in Figure 3. Higher household income, as evidenced by higher MPCE and ownership of high-value goods such as cars, computers, and washing machines, is associated with a higher probability of AC-ownership relative to EC. Residing in areas with high relative humidity, coastal areas, and urban areas is also associated with a higher probability of AC-ownership relative to EC. On the other hand, belonging to a marginalized caste is associated with a higher probability of EC-ownership. Overall, our results show that local household and district-level SEU attributes shape household-level preference for AC-versus-EC ownership and thereby enable highly context-sensitive decoupling of household-level AC-versus-EC ownership across India’s SEU-diverse districts.

**Figure 3:**
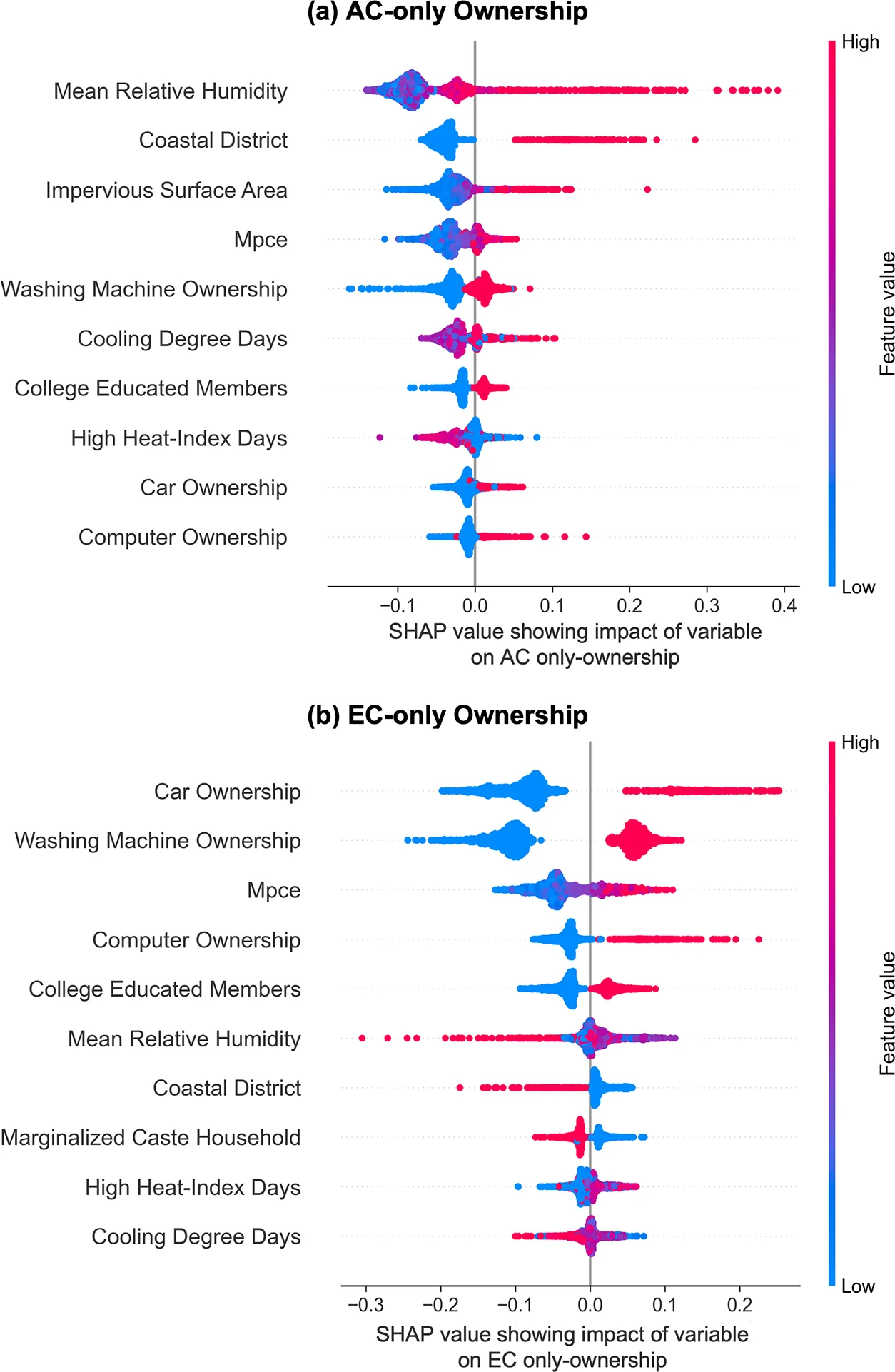
Visualizing the influence of social-environment-and-urbanization (SEU) attributes on AC-versus-EC ownership demonstrated with Shapley plots (*n* = 1500). Only the top 10 SEU attributes, excluding state effects, are shown for: (a) ownership of AC-only, (b) ownership of EC-only. Red values indicate strong influence of an SEU attribute on the outcome class; the blue values indicate low influence and can be ignored. The placement of the red values relative to the vertical line at 0 indicates the direction of influence: values to the right (positive) represent positive impact and to the left (negative) represent negative impact on the outcome class.

### 3.2 Pan-India District-Level Household-Average AC-Ownership and EC-Ownership

This is a first pan-India quantification of district-level household-average AC-versus-EC ownership (Figure 4). Across districts, AC-ownership and EC-ownership ranged between 0%–52% and 0%–93%, respectively. The median district-level household-average AC-ownership was 2%, and the median district-level household-average EC-ownership was 6.4%. The highest levels of AC-ownership (the dark blue spots in the map in Figure 4(a)) were concentrated in the biggest urban centers, such as Chennai, Ahmedabad, Delhi, and Chandigarh. High levels of EC-ownership were observed across northwestern and central India, which experience lower levels of relative humidity than southern India.

**Figure 4:**
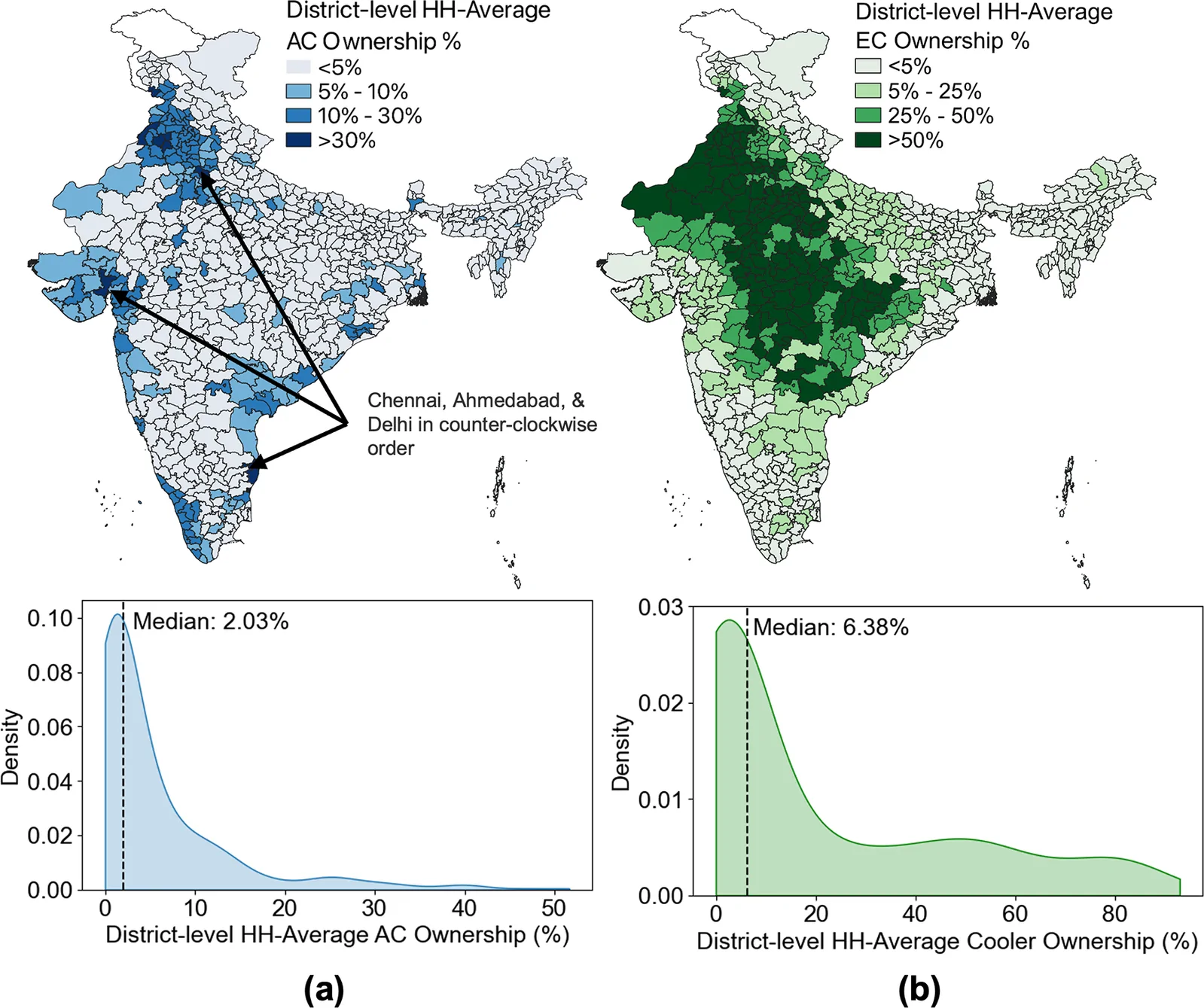
District-level household (HH)-average (a) AC-ownership % and (b) EC-ownership % spatialized from our SEU model and represented as maps with density distribution at the bottom.

Figure 5 examines district-level household-average AC-ownership relative to income (MPCE as a proxy) for two climatologically diverse states and two districts within each state, representing urban-rural variation. The relationships show a classic S-shaped curve associated with asset ownership^38^ and allow us to dive deeper into district-level household-average AC-ownership trends to demonstrate the diversity across districts. Reading down column 1 (Figure 5), Punjab, a relatively wealthy landlocked Northern state with very hot (and less humid) summers, shows 17% AC-ownership at the median MPCE (or P50_MPCE_ as noted in the figure). Chandigarh, a wealthier urban district bordering Punjab (and its capital) shows much higher ownership (53%), compared to rural Gurdaspur (5% at P50_MPCE_). In Tamil Nadu, a wealthy coastal Southern state with peak air temperatures lower than Punjab’s but much higher humidity, statewide AC-ownership is 8% at the P50_MPCE_; urban Chennai is much higher at 31%, in contrast to only 2% AC-ownership in the rural, less wealthy Thanjavur district. Figure 5 illustrates our SEU-informed AC-ownership model’s ability to account for the local SEU diversity across social, urban-rural, income, temperature–humidity, and geographic (landlocked vs coastal location) attributes when predicting district-level cooling appliance ownership across India’s 400+ districts.

**Figure 5:**
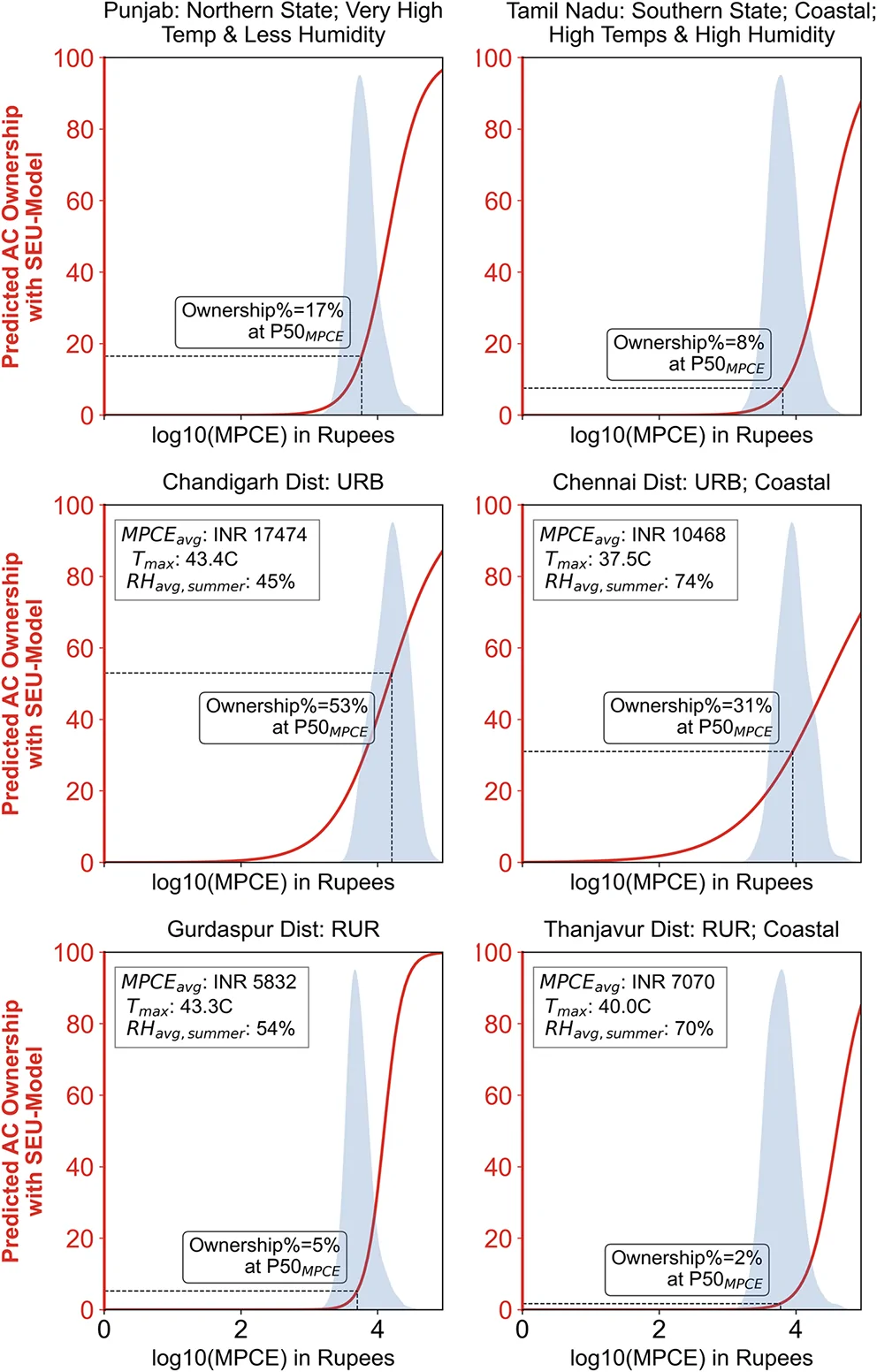
SEU-modeled AC-ownership (%) (from Section 3.1) plotted vs monthly per capita expenditure (MPCE) in two select states and 4 districts. Distribution of MPCE is shown in gray. Left column: Punjab (statewide), Chandigarh (urban district), Gurdaspur (rural district). Right column: Tamil Nadu (statewide), Chennai (urban district), Thanjavur (rural district).

### 3.3 Income Inequality in Electricity Consumption Associated with AC and EC Ownership

In India, as in many developing countries, ownership of appliances may not translate to full use due to economic constraints, but only a few studies have quantified it in a handful of cities.^14^ In this study, income-based differences in cooling appliance use are quantified at scale across three climate regions in India through new analysis enabled by our district-level database (Figure 6). Note that, as shown in the map (Figure 6), the three climate regions span a vast majority of India; we did not include the northern Indian Himalayan region shown in white because it is a cold-climate region. Similarly, the Northeast Indian region shown in hatched yellow is composite-climate like the North-Central Gangetic Plain; however, it has much greater relative humidity (being the wettest region in India) and so was analyzed separately.

**Figure 6:**
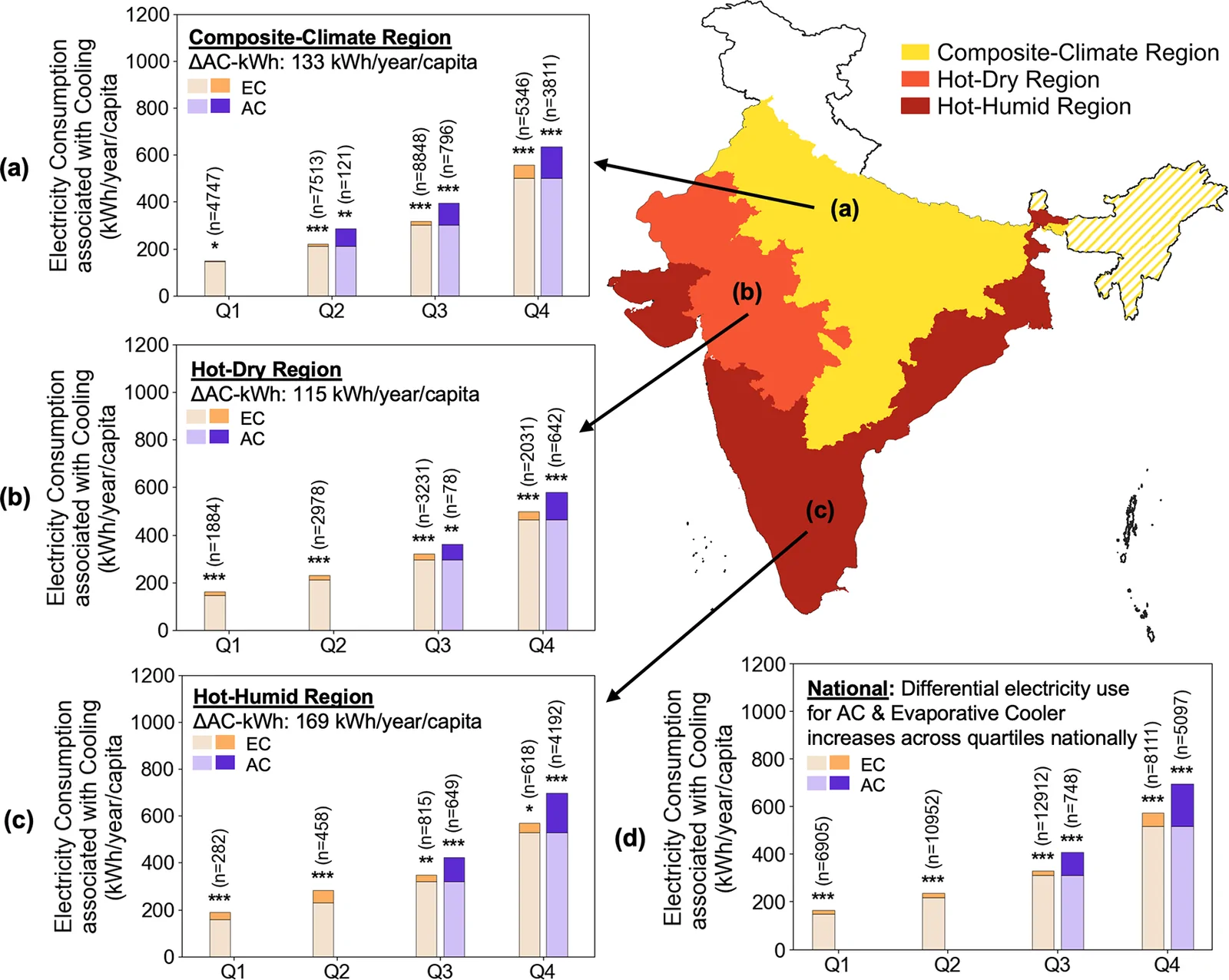
Residential electricity consumption associated with cooling appliance ownership (versus those without) across income groups (MPCE quartiles): (a) nationwide, (b) in hot-humid districts, (c) in hot-dry districts, and (d) in composite-climate districts. The bottom bar represents the average electricity consumption among homes without any cooling appliances in each quartile. Orange bars correspond to EC; purple bars correspond to AC. Sig-level: ***p < 0.001, **p < 0.01, *p < 0.05.

The differential electricity consumption associated with AC-ownership increases from lower to higher income groups—increasing from 97 (+26% higher than homes at the same income levels without ACs) to 178 kWh/capita/year (26%) nationally. In hot-humid districts, the differential ranges from 49 (18%) to 169 (25%) kWh/capita/year from MPCE Q2 to Q4; it ranges from 68 (Q3; 19%) to 115 (Q4; 17%) kWh/capita/year in hot-dry districts, and from 92 (25%) to 133 (19%) kWh/capita/year in composite-climate districts. In Northeastern districts (data not shown in Figure 6), the differential electricity consumption associated with AC-ownership is discernible only in Q4 and amounts to 181 (39%) kWh/capita/year.

As with AC-ownership, differential electricity consumption associated with EC-ownership also increases from lower to higher income groups. However, it is a fraction of the electricity consumption associated with AC-ownership. Nationally, it ranges from 15 kWh/capita/year (9% higher than homes at the same income level without ECs) to 56 kWh/capita/year (11%). In hot-humid districts, the differential ranges from 30 (17%) to 41 (6%) kWh/capita/year from MPCE Q1 to MPCE Q4. Despite the expected limited effectiveness of evaporative ECs in high-humidity regions, their use might be attributable to their being the only more affordable option, even if they are relatively ineffective compared to ACs. The differential ranges from 17 (Q1; 12%) to 35 (Q4; 8%) kWh/capita/year in hot-dry districts and from 4 (Q1; 3%) to 55 (Q4; 11%) kWh/capita/year in composite-climate districts.

Nationally, in Q4 (the highest quartile), which represents the maximum increase in AC-related electricity consumption, the increase is equivalent to a four-person home with one 1.5 kW AC operating for 5.3 h/day over the three hottest months, similar to another bottom-up study.^14^ Similarly, the differential is equivalent to 5, 4, and 4 h/day in hot-humid, hot-dry, and composite climate districts, respectively, on average among the Q4 homes. For hot-humid districts, the differential could also be construed as 2.5 h/day of AC use over half the year, given that the region experiences heat for most of the year.

## 4 Discussion

Our methodology and analyses provide key insights into who owns which type of cooling appliance, how ownership translates into use, and what inequalities in cooling appliance ownership and use exist across income groups at the district-scale pan-India for the first time.

Our paper shows that in addition to household income, local relative humidity, air temperature, urbanization, and household social characteristics play a strong role in shaping AC-versus-EC ownership. We also present novel estimates of AC-versus-EC ownership at the district-level, where most prior literature focuses on AC-ownership predominantly. Our results indicate that EC-ownership is higher on average than AC-ownership, particularly in Northwestern and Central India, but its prevalence decreases as incomes rise, in favor of AC-ownership. However, both are prevalent at low levels of ownership. And importantly, ∼75% of homes in India own neither an AC nor an EC; such data are essential to quantifying cooling inequality.

Furthermore, our analysis of differential electricity consumption for cooling shows that even when homes own ACs or ECs, they may not use them for a significant amount of time. Even in the highest-income quartile, the estimated physical use of ACs is only 4–5 h/day, assuming a 90-day hot season and 1 AC per home; this duration decreases by half in the second-highest-income group. These data, on the one hand, suggest that an increase in electricity may not occur across all of India but rather in high-income, high-ownership hotspots. At the same time, they also indicate that not only are homes without AC or EC vulnerable to heat stress (75%), but approximately half of those homes that own these appliances are not using them substantially (see Figure 6), indicating nationwide vulnerability to extreme heat, impacting almost all (90%) of India.

As India’s GDP grows, gains in household income will increase ownership of cooling appliances, but the rates will be patchy across India (see Figures 4 and 5). Overall, the low levels of cooling appliance ownership and their uneven use across income groups and climate regions (see Figure 6) point to the limits of electrically-based cooling, with ACs and ECs as private goods. This underscores the need for novel technologies, such as radiative coolers^39^ and urban design innovations, to mitigate heat hazards in India and other developing nations with similar challenges.

Overall, the SEU-informed data and methods developed in this paper can enable future electricity demand modeling and assessment of cooling poverty at the local level, accounting for local social, climate, and urbanization attributes—both of which are topics of future research in our group. The five-step methodology, developed here and tested in India, can be translated to other rapidly urbanizing developing countries where income, heat, and humidity will drive AC-ownership. Relevant survey data are available for 16 other countries, where AC-ownership has been modeled nationally,^12^ and where our approach can enable spatialization of AC/EC-ownership and use to the district level, relevant for urban-scale infrastructure future transitions modeling in several countries, especially those in the Global South, e.g., Ghana, Sierra Leone, Nigeria, South Africa, India, and Pakistan.

## 5 Limitations

Our study is based on household surveys conducted by the Government of India’s National Sample Survey Organization on Household Consumption Expenditures, and hence is limited by the limitations of the survey. Specifically, as noted in the Methods section, representativeness of survey data at the district-level is assumed given the survey’s multistage stratified sampling approach^22^ and appears to be confirmed by the observed log-normal distribution of MPCE at the district level. This study applied a minimum threshold of *n* ≥ 300 surveys to leverage the Law of Large Numbers to obtain robust estimates of AC and EC ownership. To accomplish this, 354 adjacent districts with lower sample sizes were clustered to meet this minimum threshold (out of a total of 631 original districts), of which 75% were found to have similar SEU features, indicating that clustering was largely done across adjacent districts with similar features. However, such clustering may create biases for heterogeneous districts/district-equivalents because typically smaller districts with a smaller number of surveys have now been merged. Finally, our propensity score matching analysis addresses observed confounding; however, as with any observational matching design, it cannot rule out bias from unobserved confounders, which we have attempted to quantify with Rosenbaum Bounds sensitivity analysis. It is important to keep these limitations in mind as the data and model are applied by practitioners or other scholars.

## Supplementary Material



## Notes

Data, methods and validation (Tables SI-1–4) and (Figures SI-5–7) (PDF)
