# Supplementary material for: Spatializing Air Conditioner and Evaporative Cooler Ownership, Use, and Inequality across 400+ Districts Pan-India: Unpacking Social, Environmental, and Urbanization Correlates
Source: Environ Sci Technol. 2026 Oct 7;60(43):30575–84. doi: 10.1021/acs.est.6c05101 (PMC13738697; doi:10.1021/acs.est.6c05101)

Supplementary Information (SI)

Spatializing Air Conditioner and Evaporative Cooler Ownership, Use, and Inequality across 400+ Districts pan-India: Unpacking Social, Environmental, and Urbanization Correlates

Neha Agarwal and Anu Ramaswami*

Department of Civil and Environmental Engineering & M. S. Chadha Center for Global India, Princeton University

*Corresponding Author email: anu.ramaswami@princeton.edu

Submitted to *Environmental Science & Technology* on April 6, 2026

Table of Contents

Table SI-1: Data sources for household- and district-level Social, Environmental, and Urbanization (SEU) features S2

Table SI-2 Performance of the four classification approaches, i.e., Random Forest, LightGBM, XGBoost, and Logistic Regression, tested in this paper. S3

Table SI-3: Comparison of national-level PSM results obtained from the ownership-as-treatment design with the summer-as-treatment design. S4

Table SI-4: Comparison of our estimates of different electricity consumption associated with cooling appliance use (with respect to homes without any cooling) with values reported in the literature. S5

Figure SI-5: Histogram (in grey bars, and solid line) showing near Log-normal distribution of Monthly Per Capita Expenditure (MPCE) S6

Figure SI-6: Validation of bottom-up survey-based estimates of district-average per-Capita household electricity consumption S7

Figure SI-7: Comparison of district-level household-average estimates of AC ownership (%, 2022) S8

Table SI-1: Data sources for household- and district-level Social, Environmental, and Urbanization (SEU) features

SEU Feature	Data Source	Data Granularity
Social: Household size, caste, religion, gender of head of household, presence of older (60+ years) household members, monthly per capita expenditure, asset ownership including "AC or Cooler" ownership	National Sample Survey on Household Consumption Expenditure (HCES) 2022-23	Household-level
Household Electricity Consumption in physical (kWh) units in microdata	HCES 2022-23	Household-level
Disaggregated Air Conditioner (AC) and Evaporative Cooler (EC) ownership to train Random Forest model	Consumer Pyramids Household Survey (CPS) 2022-23	Household-level
Disaggregated AC and EC ownership to validate district-level estimates	Multiple Indicator Survey 2020	Household-level
Environment: Air Temperature and Relative Humidity (for Environment)	ERA5 hourly data on single levels from 1940 to present	Gridded data at 31-km (0.25 degree) resolution (district-level climate variables obtained as average of grid cell within a district)
Urbanization: Impervious Surface Area	Global Human Settlements Layer Built Volumes dataset on Built-up Surfaces 2020	Gridded data at 1000-m resolution (district-level impervious surface area obtained as the sum of impervious surface area in all grid cells within a district)
Note: District areas ranged from 8 square kilometers to 45,000 square kilometers.		

Table SI-2 Performance of the four classification approaches, i.e., Random Forest, LightGBM, XGBoost, and Logistic Regression, tested in this paper. The overall model performance across the three Machine Learning-based classification approaches was comparable and higher than that of Logistic Regression. The uncalibrated Random Forest model was selected due to its high overall performance and high recall in the minority 'AC-only' and 'AC-plus-EC' classes shown in Figure SI-5.

Model	Balanced Acc.	Weighted F1	Macro F1	ROC-AUC (OvR)	ROC-AUC (OvO)	Cohen's Kappa
Random Forest (raw)	0.876	0.927	0.830	0.972	0.973	0.762
Random Forest (calibrated)	0.815	0.931	0.832	0.971	0.972	0.762
LightGBM (raw)	0.884	0.909	0.786	0.972	0.974	0.712
LightGBM (calibrated)	0.833	0.932	0.839	0.972	0.973	0.767
XGBoost (raw)	0.813	0.932	0.836	0.974	0.974	0.768
XGBoost (calibrated)	0.822	0.933	0.841	0.974	0.974	0.772
Logistic Regression (raw)	0.793	0.852	0.664	0.943	0.935	0.560
Logistic Regression (calibrated)	0.678	0.902	0.716	0.944	0.935	0.661

Table SI-3: Comparison of national-level PSM results obtained from the ownership-as-treatment design with the summer-as-treatment design. Only significant and robust results are reported. Estimates of differential electricity consumption associated with cooling from the former are expected to be lower than those for the latter, as the former represents effects across seasons.

Quartile	Cooling Appliance	Differential Electricity Consumption of Cooling Appliance Owners versus Non-Owners (kWh/capita/year)	Differential Summer versus Non-Summer Electricity Consumption of Cooling Appliance Owners (kWh/capita/year)
Q4	AC-Only	178	188
Q1	EC-only	15	15
Q2	EC-only	19	29
Q3	EC-only	20	75
Q4	EC-only	56	97

Table SI-4: Comparison of our estimates of different electricity consumption associated with cooling appliance use (with respect to homes without any cooling) with values reported in the literature.

**Paper**	**Scale**	**Year**	**Differential Electricity Consumption associated with Cooling Appliance Use as % Increment over Non-AC Homes**
Ramapragada et al, 2022	25 surveyed homes in Hyderabad, India	2018-19	+29% on average
Gupta et al, 2024	41 surveyed homes in Hyderabad & Jaipur, India	2018-19	+49% on average
De Cian et al, 2023	Country-level: Multiple countries including India based on nationally-pooled household survey datasets	2019	+34% on average across countries
This study	India: National across all homes and for urban-only homes; Warm-humid regions across all homes and for urban-only homes	2022-23	National: 26%-27% on average in the two highest income groups for ACs<br>Climate-Regional: 18%-26%, 10%-19%, and 19%-25% on average across income groups in hot-humid, hot-dry and composite-climate districts respectively.

Figure SI-5: Histogram (in grey bars, and solid line) showing near Log-normal distribution of Monthly Per Capita Expenditure (MPCE), used as proxy for income in this paper, for select states and districts (that are modeled in the main text). The distribution of MPCE spans a continuous range of z-scores = ±3 representing the 0.13th to the 99.87th percentiles of the distribution.

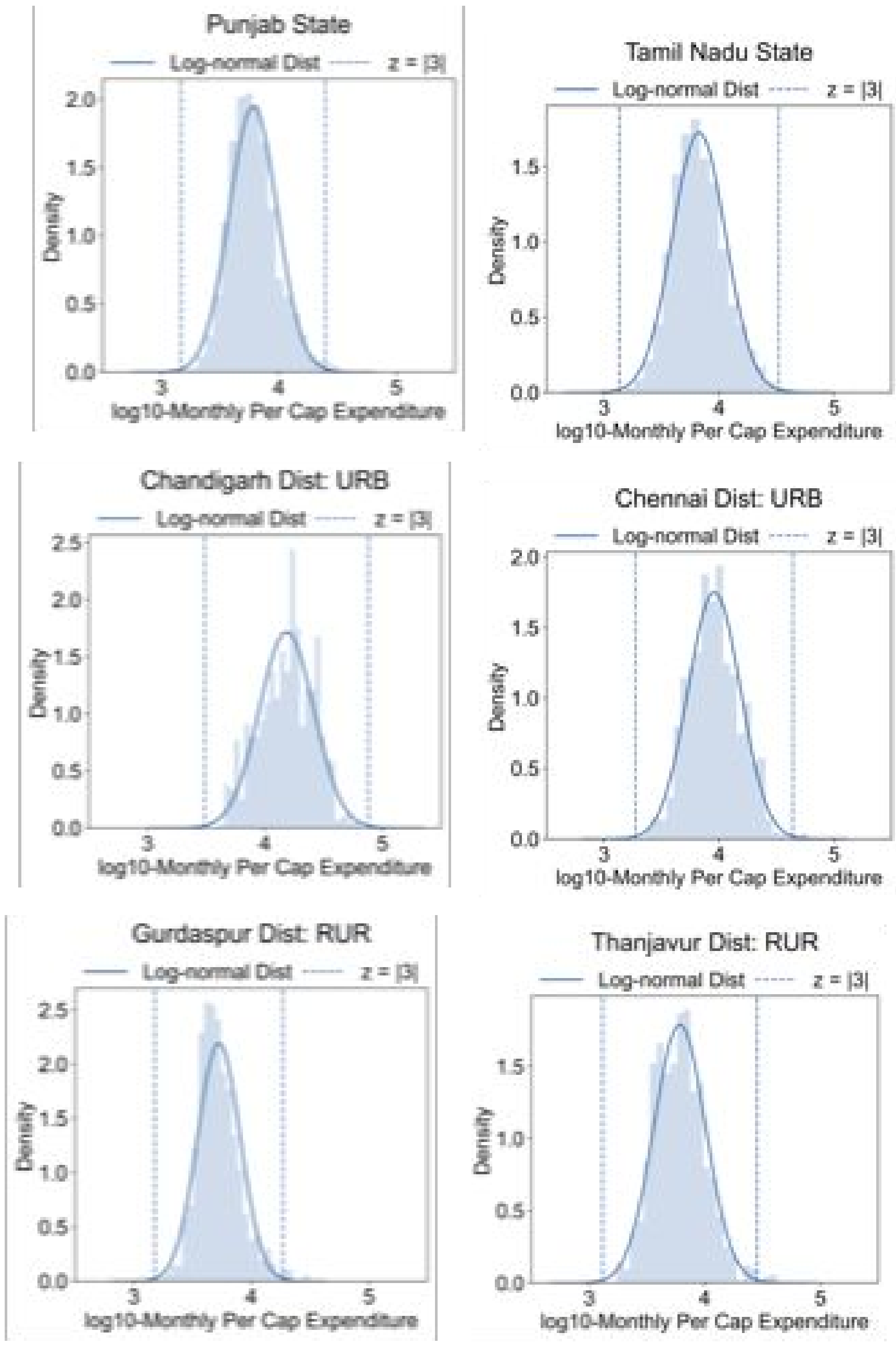

Figure SI-6: Validation of bottom-up survey-based estimates of district-average per-Capita household electricity consumption, scaled by population, with at-scale utility-reported total electricity consumption at district- and state-level: (a) based on HCES 2022 and analysis in a forthcoming paper, Ramaswami and Agarwal, Electricity Futures Modeling for all Districts pan-India, and (b) based on HCES 2011. Figure SI-6 (b) adapted from [28]. Available under a CC-BY 4.0. Copyright 2021 Tong, K., Nagpure, A.S. & Ramaswami, A.

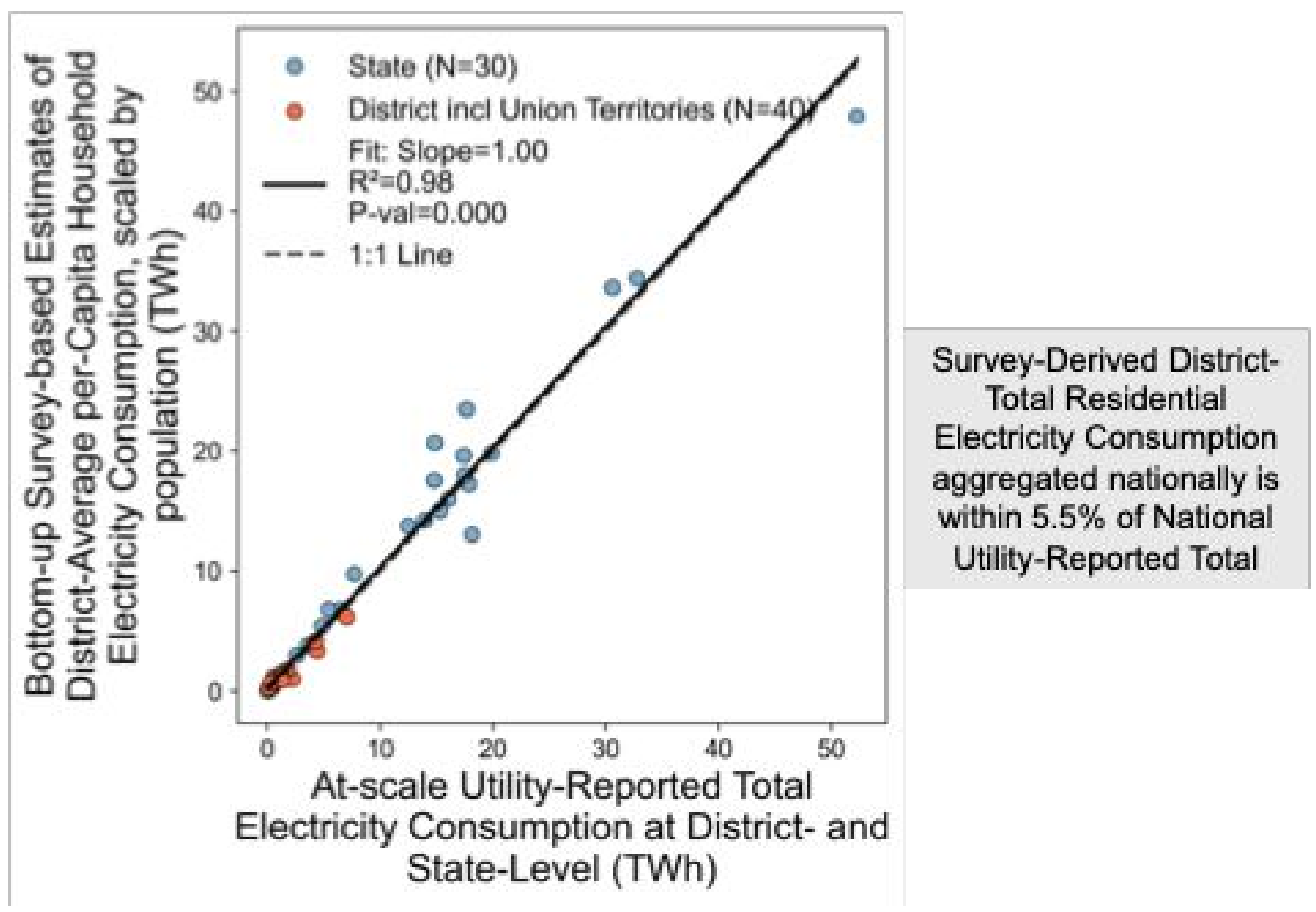

(a)

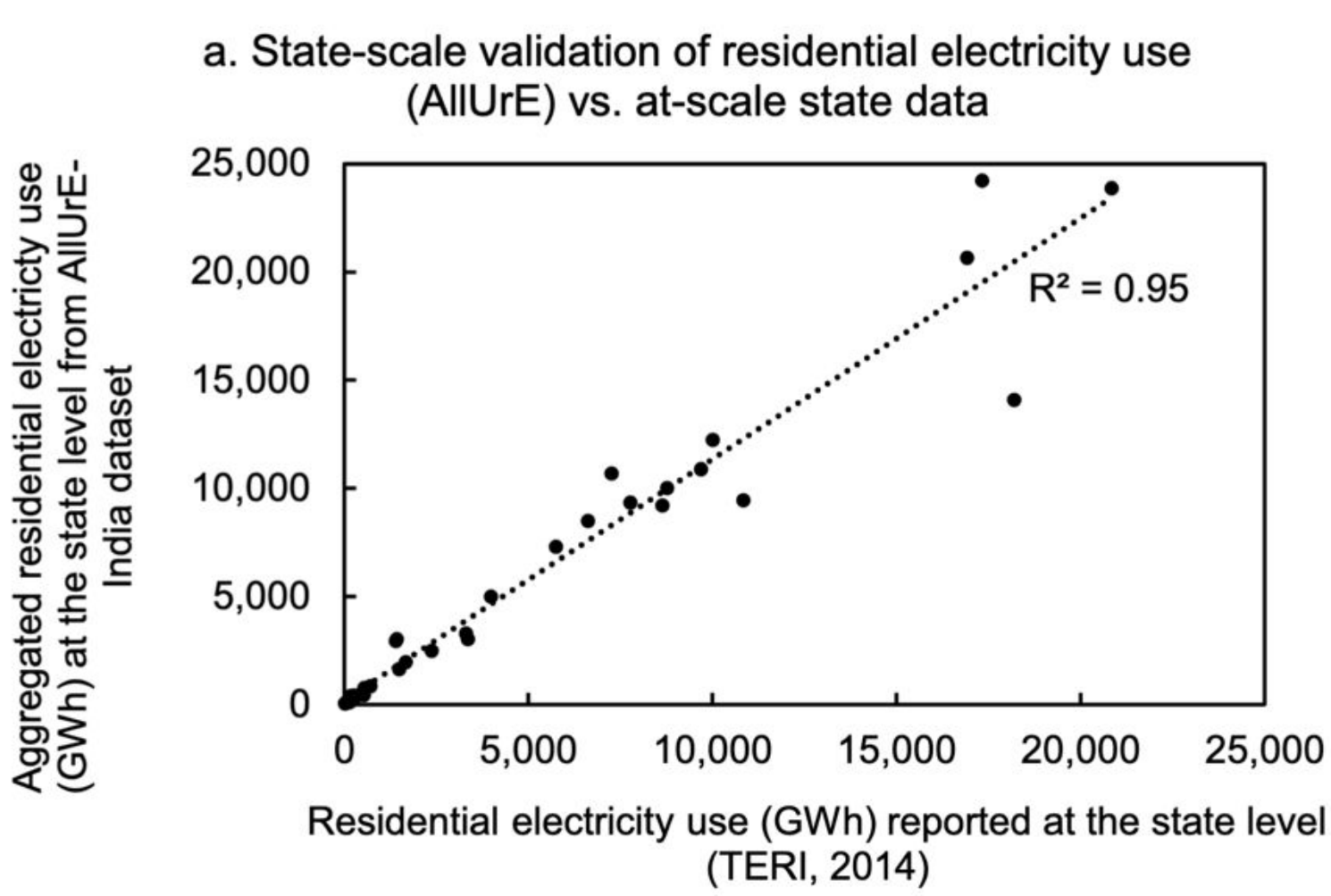

(b)

Figure SI-7: Comparison of district-level household-average estimates of AC ownership (%, 2022) from the SEU-informed model developed in this paper with AC-ownership (%, 2020 estimated from the separate MICS dataset (2020). Note that MICS is a new survey, first administered in India in 2020, and uses a different sampling strategy from HCES. The two sets of estimates show good overall agreement; we would expect estimates in the present paper for the year 2022 to be higher than in MICS 2020.

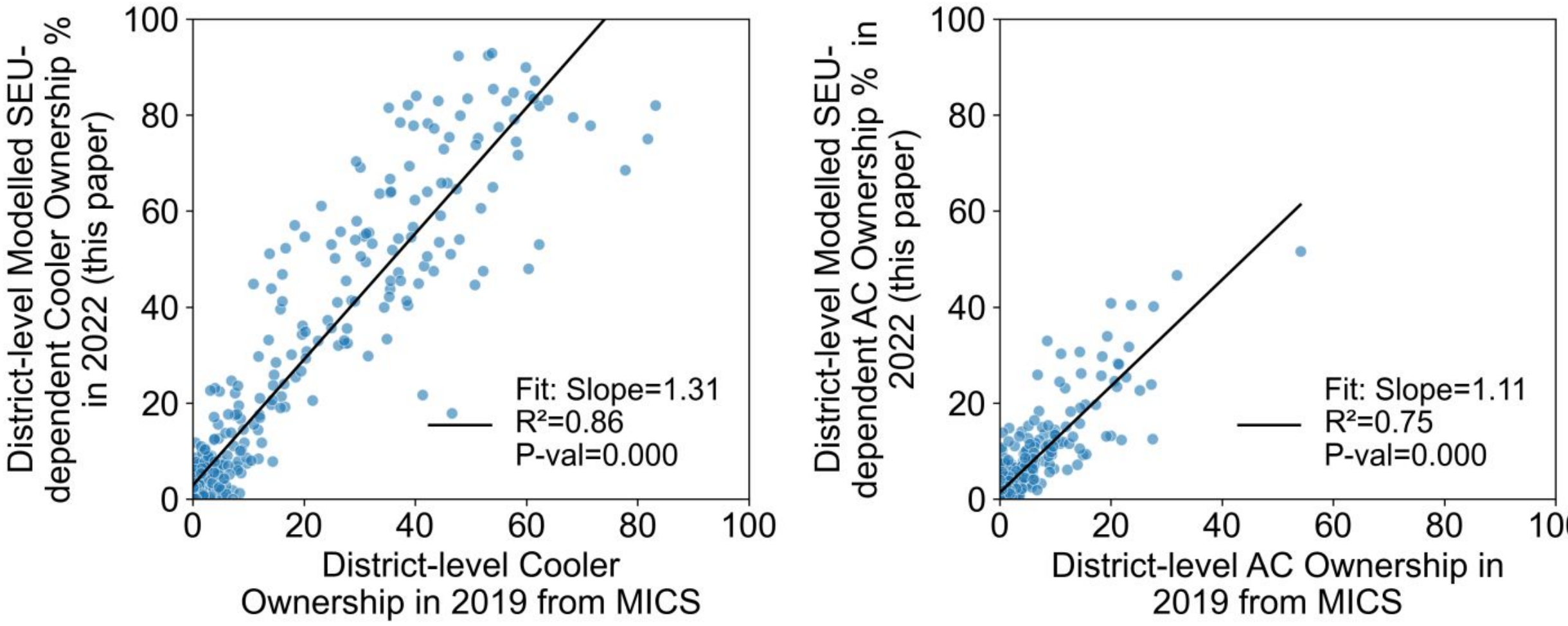

Supplement: Supplementary file 1 [file es6c05101_si_001.pdf]
